# Predicting in-hospital mortality in adult non-traumatic emergency department patients: a retrospective comparison of the Modified Early Warning Score (MEWS) and machine learning approach

**DOI:** 10.7717/peerj.11988

**Published:** 2021-08-24

**Authors:** Kuan-Han Wu, Fu-Jen Cheng, Hsiang-Ling Tai, Jui-Cheng Wang, Yii-Ting Huang, Chih-Min Su, Yun-Nan Chang

**Affiliations:** 1 Department of Emergency Medicine, Kaohsiung Chang Gung Memorial Hospital, Chang Gung University College of Medicine, Kaohsiung, Taiwan (R.O.C.); 2 Department of Computer Science and Engineering, National Sun Yat-Sen University, Kaohsiung, Taiwan (R.O.C.)

**Keywords:** Mortality prediction, MEWS, Machine learning

## Abstract

**Background:**

A feasible and accurate risk prediction systems for emergency department (ED) patients is urgently required. The Modified Early Warning Score (MEWS) is a wide-used tool to predict clinical outcomes in ED. Literatures showed that machine learning (ML) had better predictability in specific patient population than traditional scoring system. By analyzing a large multicenter dataset, we aim to develop a ML model to predict in-hospital morality of the adult non traumatic ED patients for different time stages, and comparing performance with other ML models and MEWS.

**Methods:**

A retrospective observational cohort study was conducted in five Taiwan EDs including two tertiary medical centers and three regional hospitals. All consecutively adult (>17 years old) non-traumatic patients admit to ED during a 9-year period (January first, 2008 to December 31th, 2016) were included. Exclusion criteria including patients with (1) out-of-hospital cardiac arrest and (2) discharge against medical advice and transferred to other hospital (3) missing collect variables. The primary outcome was in-hospital mortality and were categorized into 6, 24, 72, 168 hours mortality. MEWS was calculated by systolic blood pressure, pulse rate, respiratory rate, body temperature, and level of consciousness. An ensemble supervised stacking ML model was developed and compared to sensitive and unsensitive Xgboost, Random Forest, and Adaboost. We conducted a performance test and examine both the area under the receiver operating characteristic (AUROC) and the area under the precision and recall curve (AUPRC) as the comparative measures.

**Result:**

After excluding 182,001 visits (7.46%), study group was consisted of 24,37,326 ED visits. The dataset was split into 67% training data and 33% test data for ML model development. There was no statistically difference found in the characteristics between two groups. For the prediction of 6, 24, 72, 168 hours in-hospital mortality, the AUROC of MEW and ML mode was 0.897, 0.865, 0.841, 0.816 and 0.939, 0.928, 0.913, 0.902 respectively. The stacking ML model outperform other ML model as well. For the prediction of in-hospital mortality over 48-hours, AUPRC performance of MEWS drop below 0.1, while the AUPRC of ML mode was 0.317 in 6 hours and 0.2150 in 168 hours. For each time frame, ML model achieved statistically significant higher AUROC and AUPRC than MEWS (all *P* < 0.001). Both models showed decreasing prediction ability as time elapse, but there was a trend that the gap of AUROC values between two model increases gradually (*P* < 0.001). Three MEWS thresholds (score >3, >4, and >5) were determined as baselines for comparison, ML mode consistently showed improved or equally performance in sensitivity, PPV, NPV, but not in specific.

**Conclusion:**

Stacking ML methods improve predicted in-hospital mortality than MEWS in adult non-traumatic ED patients, especially in the prediction of delayed mortality.

## Introduction

In an era of increasing numbers of patients and limited hospital resources, methods to reduce uncertainty are particularly warranted in the fast-paced, chaotic environment of the emergency department (ED) (Croskerry, 2013). The ED faces substantial challenges in the early identification of patients whose condition might deteriorate if optimal and timely critical care is not provided. Failure to recognize and appropriately manage patients with deteriorating conditions is associated with higher in-hospital mortality rates and costs (Fernando et al., 2018; Chen et al., 2015) and may result in legal and patient safety concerns. Thus, feasible and accurate risk prediction systems for ED patients are urgently needed.

Traditional rule-based scoring systems based on risk factor investigation and regression models have been developed to predict catastrophic events, such as cardiac arrest or death. Since its introduction in 2001 by Subbe and colleagues (Subbe et al., 2001), the modified early warning score (MEWS) has been widely used to predict clinical outcomes. MEWS was calculated using five simple physiological parameters (systolic blood pressure (SBP), pulse rate (PR), respiratory rate (RR), body temperature (BT), and level of consciousness) that can easily be obtained through the electronic triage system. Previous studies have reported that MEWS can help predict mortality (Subbe et al., 2001; Churpek et al., 2014; Dundar et al., 2016; Xie et al., 2018; Klug et al., 2020; Mitsunaga et al., 2019; Ong et al., 2012; Bulut et al., 2014; Churpek et al., 2017). However, the prediction performance varied and was inadequate, with an area under the receiver operating characteristic curve ranging from as low as 0.630 for general medical and surgical ED patients to the highest value of 0.891 for ED patients aged >65 years.

As an alternative to traditional scoring systems, artificial intelligence has been introduced in the ED clinical field for different aspects, including risk stratification and complication prediction (Grant et al., 2020). Previous studies have shown that machine learning (ML) techniques may effectively predict several adverse events, such as cardiac arrest, shock, mechanical ventilation, and in-hospital mortality (Ong et al., 2012; Grant et al., 2020; Taylor et al., 2016; Gutierrez, 2020; Kim et al., 2019; Yee et al., 2019; Kwon et al., 2018a). ML also showed better predictability for specific patient populations than certain validated clinical decision rules in wards (Churpek et al., 2016; Mohamadlou et al., 2019; Kwon et al., 2018b; Lee et al., 2018; Kia et al., 2020) or intensive care units (ICUs) (Kim et al., 2019; Yee et al., 2019; Desautels et al., 2016). For general ED populations, two single-center studies showed that ML models surpassed the traditional scoring system in predicting early (<48 h) and short-term mortality (2–30 days) (Klug et al., 2020), as well as cardiac arrest, within 24 h (Jang et al., 2019). Using a large Korean national dataset, Kwon et al. (2018a) showed that the deep-learning-based triage system significantly outperformed MEWS in overall in-hospital mortality (0.935 *vs.* 0.810) prediction. The ML algorithms may be advantageous in creating new models for predictive analysis in emergency care.

By analyzing a large multicenter dataset, the objective of this study was to develop an ensemble stacking ML model to predict in-hospital mortality for adult non-traumatic ED patients at different time points and compare the prediction performance with MEWS and other ML models. We believe that the stacking ML models can achieve better prediction results and may reduce uncertainty in the chaotic, fast-paced ED environment.

## Materials & Methods

### Study Design

This was a retrospective observational cohort study. The study adhered to the Transparent Reporting of a multivariable prediction model for individual prognosis or diagnosis (TRIPOD) statement regarding the reporting of predictive models. The Chang Gung Medical Foundation approved this research and waived the need for patient consent (201801637B0).

### Study Setting and Population

Based on the chief complaint and patients’ condition, the triage nurse evaluated all ED patients and then divided them into trauma or non-trauma categories in the electronic chart system. The study population consisted of consecutive adult (aged >17 years) non-traumatic patients admitted to the ED for 9 years (January 1, 2008, to December 31, 2016). Exclusion criteria included patients with the following conditions: (1) out-of-hospital cardiac arrest and death upon arrival; (2) inability to receive follow-up examinations, owing to discharge against medical advice or transfer to another hospital from the ED; and (3) missing variables in the electronic database. We excluded patients with out-of-hospital deaths because their high mortality rates could falsely improve the performance of the prediction models.

The study sites included five Taiwanese EDs that belonged to a single healthcare system and were geographically dispersed nationwide. The two EDs were tertiary referral medical centers with over 3500 and 2500 beds, respectively. The other three were secondary regional hospitals with more than 1200, 1000, and 250 beds each. The cumulative mean annual ED visits in the five EDs were over 480,000, and the annual health insurance claim costs of the studied hospitals accounted for approximately 10% of the national health budget according to government statistics. All EDs use the same healthcare informatics system with a centralized data warehouse.

### Outcome measures and other variables

For all analyses, the primary outcome was in-hospital mortality, which was categorized as imminent mortality (≤ 6 h after ED registration), early mortality (≤ 24 h after ED registration), mid-term mortality (≤ 72 h), and delayed mortality (≤ 168 h). A six-hour prediction window was chosen based on clinical considerations that the typical duration of physician shift length (8–12 h) and the desire to alert the same care team (Kia et al., 2020). The 24-and 72-hour predictions were analyzed to help clinicians accurately dispose of intensive care unit admission. Patients who died over 168 h were included in the study group. All data elements for each ED visit were obtained from the electronic chart database of the central hospital. Our study attempted to develop a model using only simple clinical traits that could be obtained during triage. Therefore, only variables available at the time of triage were collected, including demographic information, time of ED visit, triage vital signs including BT, PR, RR, and level of consciousness using the Glasgow Coma Scale (GCS). The time of death was also recorded if in-hospital mortality occurred. MEWS was calculated as a total of five subcomponent scores (Table 1). The component for the level of consciousness was defined using the AVPU score (A for alert, V for reacting to vocal stimulus, P for reacting to pain, and U for unresponsive) in MEWS. Therefore, we converted GCS to AVPU as follows: 13–15 GCS points as alert; 9–12 GCS points as voice; 4–8 GCS points as pain; ≤ 3 GCS points as unresponsive, based on previous studies (Mohamadlou et al., 2019).

### ML model design

**Figure 1:**
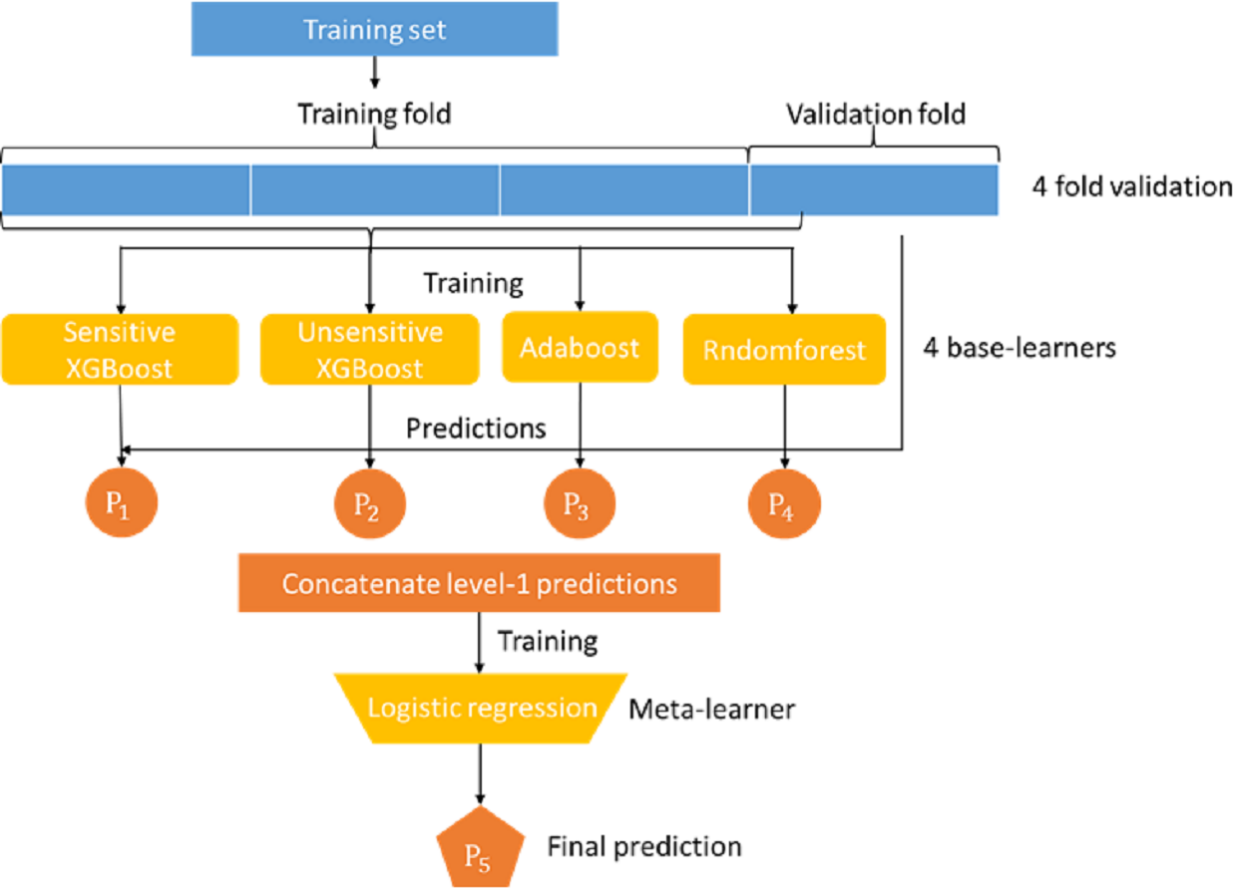
Proposed stacking model for predicting in-hospital mortality for adult non-traumatic emergency department patients.

Mortality prediction was associated with the supervised ML models. Many ML models can be used for this application, such as logistic regression, decision trees, and support vector machines. In this study, we developed an ensemble stacking model by combining several powerful base learners, including sensitive Xgboost, insensitive Xgboost, random forest, and Adaboost. Each of these base learners is sufficiently powerful to provide a good prediction. However, by stacking these base learners together *via* a meta-classifier, the ensemble model can be expected to deliver better and more stable prediction results, (Rokach, 2009) as shown in the Figure model (Fig. 1), where the logistic regression model serves as the final meta-classifier to merge the results of four base learners. The base learners adopted in our stacking model are described as follows:

### XGBoost

XGBoost [xgboost] belongs to the family of gradient boosted algorithms, but can be regarded as an upgrade of the gradient boosted decision tree (GBDT) (Chen & Guestrin, 2016). This algorithm applies the Taylor series to approximate the objection. The first-order and second-order derivatives of the objective functions are derived and used to guide the construction of a series of decision trees. In addition to the new approximation of the object function, in order to avoid over-fitting, the regularization term is also added to the loss as shown in Eq. (1), where *T*_*k*_ and *w*_*k*,*j*_ represent the number of leaf nodes and the weight of leaf node of the jth node of the kth tree, respectively. The notation *f*_*k*_(*x*_*i*_) represents the inference output of the kth tree for the ith input data. Similar to many other models used for classification, Xgboost can be tuned through a hyper-parameter denoted as ***scale_pos_weight*** to differentiate the weights of positive and negative samples. This tuning is very important for an imbalanced dataset. In this study, the number of positive cases was much lower than the number of negative cases. Therefore, our stacking model included two XGBoost models denoted as sensitive XGBoost and insensitive XGBoost, both of which put more weight on positive samples by setting ***scale_pos_weight*** to $1.5\times \left( \frac{\mathrm{Nn}}{\mathrm{Np}} \right) $ and $0.85\times \left( \frac{\mathrm{Nn}}{\mathrm{Np}} \right) $, respectively, where Np and Nn represent the number of positive and negative samples, respectively. Using these two Xgboost models with different weight settings can increase the diversity of the base learners in our stacking model and help the overall model to achieve better sensitivity.

(1) \begin{eqnarray*}Loss={\mathop{\sum \nolimits }\nolimits }_{i=1}^{n}cross\text{_}entropy \left( {y}_{i},{\mathop{\sum \nolimits }\nolimits }_{k=1}^{200}{f}_{k}({x}_{i}) \right) +{\mathop{\sum \nolimits }\nolimits }_{k=1}^{200}(\gamma {T}_{k}+\lambda {\mathop{\sum \nolimits }\nolimits }_{j=1}^{{T}_{k}}{w}_{k,j}^{2}).\end{eqnarray*}

**Table 1:** Modified early warning score.

Score	3	2	1	0	1	2	3
Systolic blood pressure (mm Hg)	<70	71–80	81–100	101–199		≧200	
Heart rate (bpm)		<40	41–50	51–100	101–110	111–129	≧130
Respiratory rate (bpm)		<9		9–14	15–20	21–29	≧30
Temperature (C)		<35		35–38.4		≧38.5	
Level of Conscious (AVPU)				Alert	Reactive to Voice	React to Pain	Unresponsive

### AdaBoost

AdaBoost (adaptive boosting) is another boosting algorithm that combines multiple weak classifiers into a strong one (Freund & Schapire, 1997). The type of classifier used in AdaBoost is a decision tree, and the loss function used is shown in Eq. (2), where ${f}_{k} \left( . \right) $ and *α*_*k*_ represent the function and weight of each tree, respectively. A total of 150 trees were used in the AdaBoost model.

(2) \begin{eqnarray*}Loss=\exp \nolimits (-{\mathop{\sum \nolimits }\nolimits }_{i=1}^{n}{y}_{i}{\mathop{\sum \nolimits }\nolimits }_{k=1}^{150}{\alpha }_{k}\times {f}_{k}({x}_{i})).\end{eqnarray*}

### Random forest

Random forest (RF) is a combination of multiple decision trees. It randomly samples training data and features to train and build each sub-tree (Ho, 1995). Ideally, each tree can learn something different from the others. The final prediction results of the random forest were generated by unifying the prediction results of all trees through averaging or voting. The random forest used in our stacking model consisted of 150 trees. The loss function adopted in our random forest model is cross-entropy. The class weight parameter was set to ***balance***, which automatically sets the weight of each class equal to the reciprocal of its class sample size.

Because the meta-learner and the base learners belong to different types of models that require different training methods, they cannot be trained in a single phase. The base learners must be trained first, and then the meta-learners can be trained with the output of the base learners. In addition, the data used to train the base learners cannot be applied to the base learners to generate the training data of the meta learner because this will likely lead to overfitting. Therefore, we adopted the following steps to train our stack model:

1. The dataset was first randomly split into 67% training and 33% test data. The training data were further split into k-folds (*k* = 4 in this study).
2. Choose k-1 folds of data to train the base classifier first.
3. Apply the remaining one fold to each individual trained base model to obtain the prediction result of each model.
4. Use the inference results of base models *P*_1_, *P*_2_, *P*_3_, and *P*_4_ to train the meta learner model.
5. Repeat steps 2-5 for different fold combinations of data.

Overall, steps 2-5 were run for k rounds.

### Data analysis

Continuous variables are presented as means (standard deviation) or medians (interquartile range) and were analyzed using a two-tailed Student’s *t*-test. The normality of continuous variables was examined using the Kolmogorov–Smirnov test. Categorical variables were presented as numbers (percentages) and were analyzed using the chi-squared test. The proposed stacking model adopted features (BT, HR, BP, RR, and GCS) used in the MEWS scoring system plus two additional features of age and sex. We conducted a performance test (sensitivity, specificity, positive predictive value (PPV), and negative predictive value (NPV)) of the MEWS and ML methods for predicting in-hospital mortality at 6, 24, 72, and 168 h. We also examined the area under the receiver operating characteristic curve (AUROC) and the area under the precision and recall curve (AUPRC) as comparative measures.We also calculated the performance of the stacking model by adopting only the variable used in the MEWS scoring system for comparison. The ROC curve represents the tradeoff between the sensitivity and specificity of a model, and AUROC is an overall metric for evaluating ROC performance. However, with imbalanced data, in which the number of negatives outweighs the number of positives, it is still likely that both sensitivity and specificity values are all high. In other words, the ratio of false-positive cases over the entire actual negative cases is low, but its number is not small compared with the true positive cases, such that the model cannot distinguish between true and negative-positive cases. Therefore, AUPRC is a more suitable metric for evaluating model performance (Kwon et al., 2018a), and the comparison of AUROC and AUPRC was performed using the ANOVA test. The AUPRC curve is created by plotting the precision and the recall rate for various thresholds. Here we only consider those thresholds which will lead to either non-zero true positive (TP) or non-zero false positive (FP) values. The edge cases where both TP and FP are zero are neglected since the precision value cannot be calculated. Therefore, the starting precision values of the PRC curves are not fixed.

All data were stored in Excel (Microsoft Office 2007; Microsoft, Redmond, WA, USA) and imported into SPSS software (version 17.0; SPSS Inc., Chicago, IL, USA) and STATA software (version 11.1; STATA Corporation, College Station, TX, USA) for statistical analysis.

## Results

**Figure 2:**
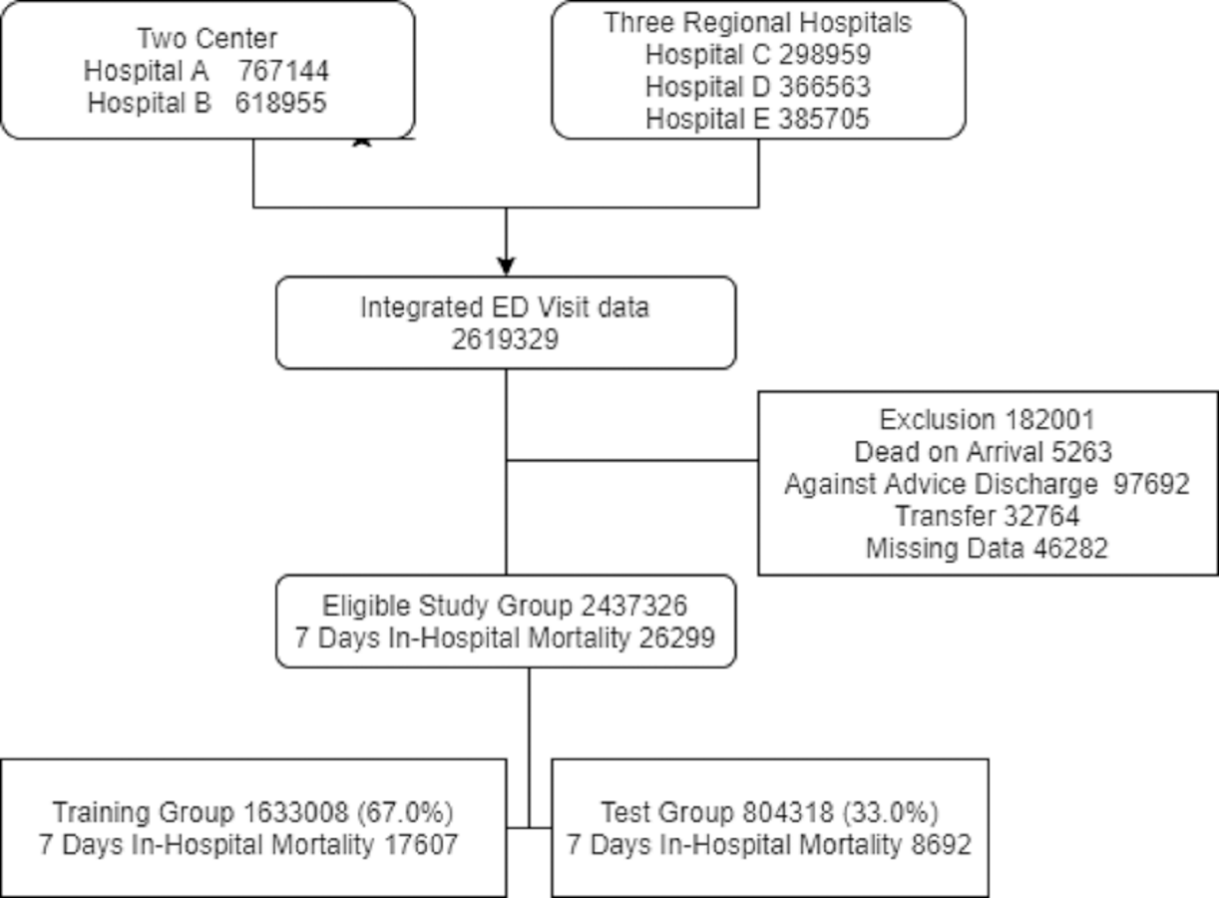
The inclusion and exclusion of study population.

Between 2008 and 2016, there were 2,619,329 ED visits in the five study hospitals. We excluded 182,001 visits, as 5263 patients died upon arrival, 32,764 were transferred from the ED, 97,692 were discharged against medical advice, and 46,282 had missing data. The study group consisted of 2,437,326 ED visits and 26,299 in-hospital deaths (1.07%) that occurred within 7 days after the ED visit (Fig. 2). Table 2 shows the baseline and clinical characteristics of ED visits. Variables including age, sex, and vital signs were all normally distributed, and there was no statistically significant difference in the characteristics between the training group (1,633,008, 67.0%) and test group (804,318, 33.0%).

**Table 2:** Baseline and clinical characteristics of the study group.

Variables	Study group	Training group	Test group	*p* value
	2437326	1633008 (67.0%)	804318(33.0%)	
Age (years) (Mean ± SD)	54.33 ± 19.72	54.31 ± 19.72	54.35 ± 19.71	0.1382
Gender, Male (N, %)	1208337 (49.57)	809316 (49.56)	399021 (49.61)	0.4627
Initial Vital sign				
SBP	140 (122–160)	140 (122–160)	140 (122–160)	0.8774
DBP	82 (72–94)	82 (72–94)	82 (72–94)	0.6005
RR	18 (18–20)	18 (18–20)	18 (18–20)	0.1260
HR	87 (75–101)	87 (75–101)	87 (75–101)	0.2223
Temperature	36.5 (36.1–37)	36.5 (36.1–37)	36.5 (36.1–37)	0.2089
Initial GCS (N, %)				
13–15	2309893 (94.77)	1547758 (94.78)	762135 (94.76)	0.8356
9–12	76631 (3.14)	51285 (3.14)	25346 (3.15)	
4–8	40649 (1.67)	27194 (1.67)	13455 (1.67)	
≦3	10153 (0.42)	6771 (0.41)	3382 (0.42)	
Disposition (N, %)				
discharged	1772841 (72.7)	1187816 (72.7)	585025 (72.7)	0.566
ICU admission	70978(2.9)	47672(2.9)	23306(2.9)	
Ward admission	583296(23.9)	390633(23.9)	192663(24.0)	
Mortality (N, %)					
≦6 h	3163(0.13)		2137(0.13)		1026(0.13)		0.5008	
≦24 h	8613(0.35)		5818(0.36)		2795(0.36)		0.2776	
≦72 h	17057(0.69)		11436(0.70)		5621(0.70)		0.8984	
≦168 h	26299(1.07)		17607(1.08)		8692(1.08)		0.8605	

**Notes.**

IQR interquartile range SBP systolic blood pressure DBP diastolic blood pressure HR heart rate RR respiratory rate BT body temperature ICU intensive care unit

**Figure 3:**
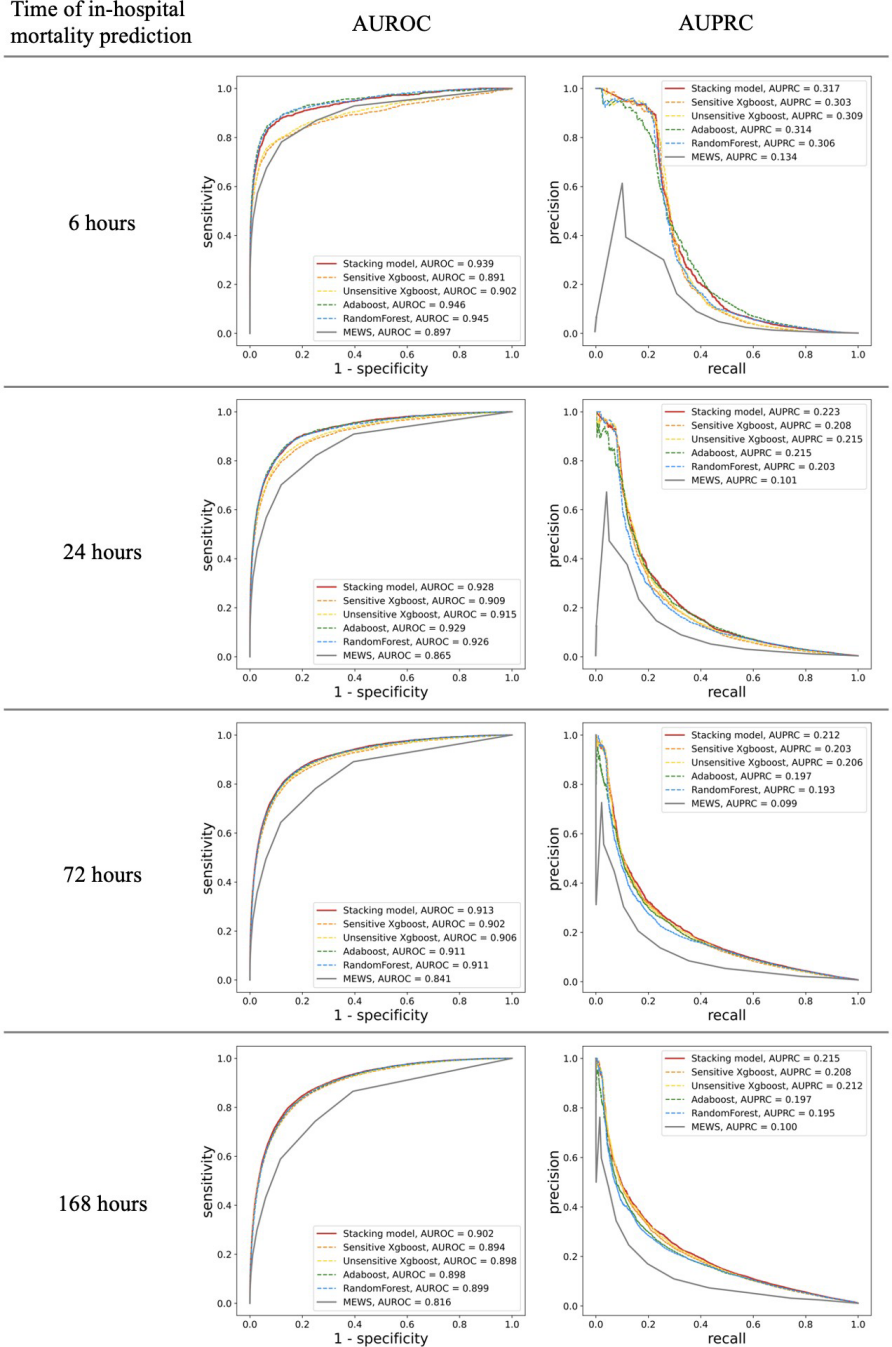
Comparison of accuracy for predicting in-hospital mortality in each time frame. Shown by area under the receiver operating characteristic (AUROC) and the area under the precision and recall curve (AUPRC).

MEWS showed good performance for in-hospital mortality prediction, as its AUROC values were up to 0.897 (95% confidence interval [CI] 0.886–0.908) and 0.816 (0.812–0.822) for the 6-h and 168-h in-hospital mortalities, respectively. The stacking ML model showed better performance with AUROC values of 0.939 (0.931–0.946) and 0.902(0.898–0.905) for the 6-h and 168-h in-hospital mortalities, respectively. In addition, the AUPRC values of MEWS for different time stages were low and all <0.15 (0.134 for 6 h and 0.100 for 168 h). For the prediction of in-hospital mortality over 48 h, the AUPRC performance dropped below 0.1, while that of the ML model was 0.317(0.289) for 6 h and 0.215(0.205–0.224) for 168 h. For every time frame, our stacking model achieved higher AUROC and AUPRC values than the predictive performance of MEWS (Fig. 3) (difference in AUROC, all *P* < 0.001). In particular, the AUPRC values of the proposed ML model are more than twice those of MEWS. Both models showed a decrease in predictive ability over time. However, there was a significant trend in which the gap in AUROC values between the stacking ML model and MEWS increased gradually (*P* < 0.001). The performance of the proposed ML model using only the variable used in the MEWS scoring system could still outperform MEWS in the AUROC metric (0.933 for 6 h and 0.876 for 168 h) and AUPRC (0.316 for 6 h and 0.210 for 168 h). Figure 3 shows the ROC and PRC curves for each base model. The AUROC performances of each ML model were very similar. However, the stacking model had the best AUPRC values compared with each individual model for all four time periods.

**Figure 4:**
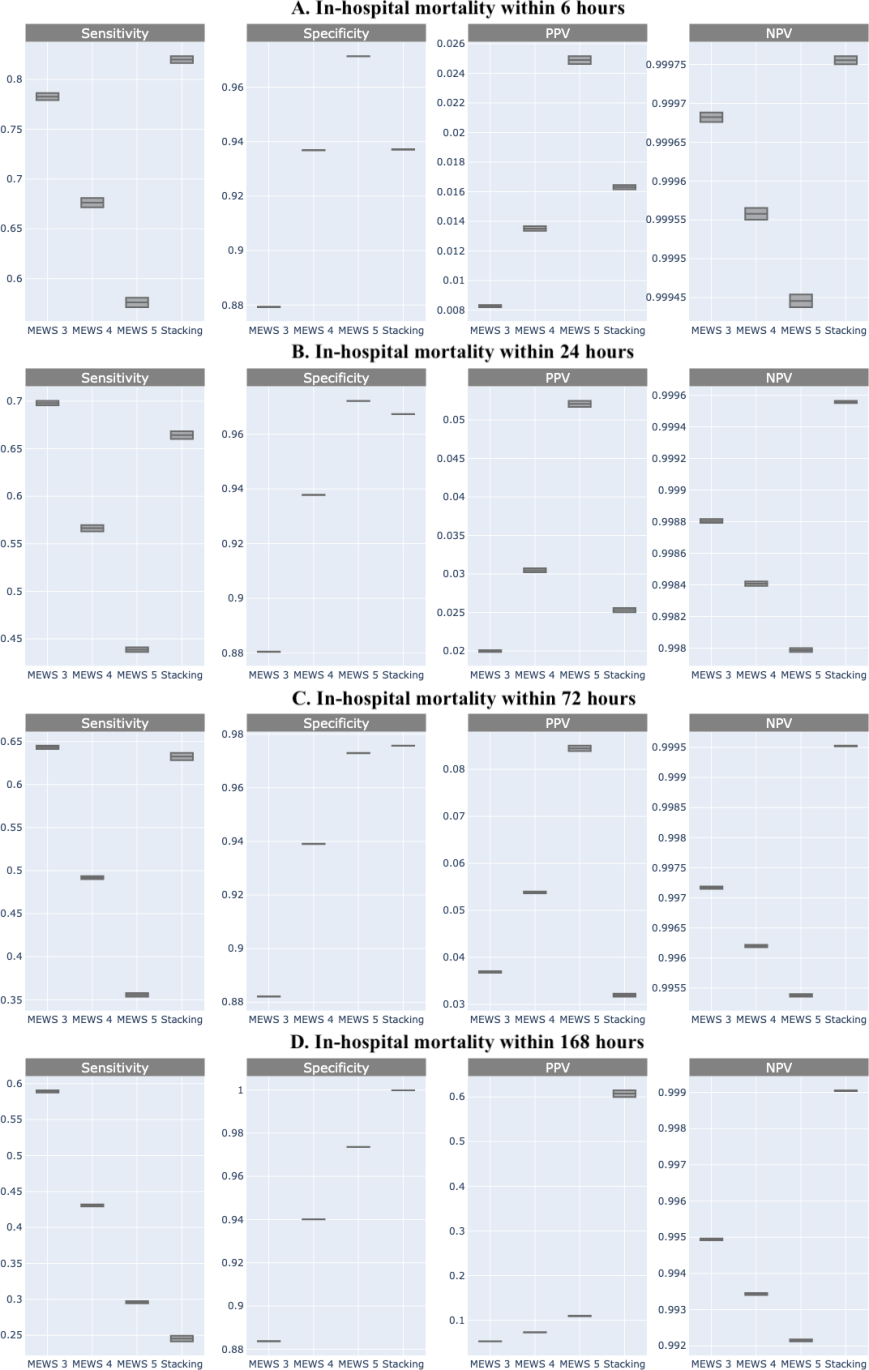
Comparison of sensitivity, specificity, positive predictive value, and negative predictive value of machine learning and Modified Early Warning Score (MEWS) in each time frame. (A) In-hospital mortality within 6 h; (B) In-hospital mortality within 24 h; (C) In-hospital mortality within 72 h; (D) In-hospital mortality within 168 h.

Comparing the predictive performance (sensitivity, specificity, and predictive values) of in-hospital mortality at different times required a fixed threshold level for each classifier. It can be derived from the ROC curve of MEWS-based prediction that the optimal cut-off point is 3.07. Because MEWS can only be an integer, based on this optimal point, patients whose MEWS value is larger than 3 will be classified as positive. In addition to this optimal cut-off point (MEWS >3), we also provide the results for the other two MEWS thresholds (score >4 and >5). Compared with MEWS, the stacking ML model consistently showed improved or equal performance in sensitivity, PPV, and NPV, but not in specificity (Fig. 4).

## Discussion

In this large, multicenter cohort study, we found that the MEWS showed good performance. However, the ML model was slightly better at predicting in-hospital mortality in adult non-traumatic ED patients. Ong et al. (2012) first reported the potential of the ML model incorporating vital signs and heart rate variability parameters for better predictability (AUROC for the ML model is 0.741 compared with 0.693 for MEWS), more specifically, for predicting mortality in high-acuity ED patients. For adult ED patients, a model that utilized the nine features combined with vital signs, chief compliances, and emergency severity index yielded an AUC of 0.962 for early mortality (48 h) and 0.923 for short-term mortality (2–30 days) (Klug et al., 2020). A deep-learning-based triage system developed based on age, sex, chief complaint, the period between symptom(s) onset and ED visit, arrival mode, presence of trauma, initial vital signs, and mental status as input parameters also showed convincing predictive ability regarding in-hospital mortality (0.935 *vs.* 0.810) (Kwon et al., 2018a). Using the more simplified input parameters easily obtained at triage, our studies showed similar performance with an AUROC of 0.951 for 6 h and 0.908 for 7 d after presentation at the ED.

The input parameters used in our study included only seven structured variables (age, sex, vital signs (BT, BP, HR, RR), GCS) available during the ED triage, which has several advantages. First, these basic and clear-cut data do not require expert judgment and have low inter-health care provider variations. Second, the time of symptom onset to ED arrival data was not used, thereby potentially avoiding the influence of patient statements. Patients typically could not identify the specific time of symptom onset, and the data might be misleading. Third, models that used hundreds of variables made manual entry impractical and were associated with increased difficulty in encoding databases. A simplified model with feasible and reliable input parameters can be efficiently collected in a variety of clinical settings. Indeed, it would be of great value in a resource-constrained ED setting, thus increasing the possibility of implementation in other settings.

Another finding of our study was the identification of predictive performance at each time stage. MEWS showed the highest ability to predict the 6-h in-hospital mortality (AUROC: 0.897) but quickly declined as the AUROC for the 7-d in-hospital mortality prediction was only 0.816. Examination of the stacking model showed slightly better results in predicting the 168-h in-hospital mortality. Overall, the sensitivity of the ML model was higher than that of the MEWS model, without lowering the specificity and PPV. In addition, the AUPRC of the proposed ML model was twice that of MEWS. Although the stacking model only marginally improved the predictability, small improvements in accuracy for mortality prediction could still have benefits. It is estimated that for ten thousand patients, the stacking model can correctly predict approximately 18.4 more deaths among 127.5 deaths than MEWS for the 6-h mortality prediction, with fewer false-positive predictions than MEWS, at a threshold score of 4. For long-term predictions, such as 168 h, the stacking model can predict 410 more deaths among 10,80 deaths than MEWS, although at the expense of 100,00 or more false-positive predictions. Given the statistically significant differences in our study, the stacking model could potentially be more useful in EDs than in MEWS.

In this study, an ensemble stacking model consisting of four base learners, including sensitive Xgboost, insensitive Xgboost, random forest, and Adaboost, was developed for mortality prediction. The base models adopted are strong classifiers. Each individual model is strong enough to deliver very good AUROC performance, such that the stacking of these models can hardly improve the AUROC metric further. In fact, due to the data imbalance nature in our study, even MEWS can achieve AUROC no less than 0.8, for all time periods. This might be the reason our stacking model only slightly improves the performance of single models, because the ML models tested in this study may closely reach the maximum obtainable prediction capability from the available features of the patients. However, as mentioned before, AUPRC is a better performance indicator for imbalanced data, and all four base learners and our stacking models showed that the ML technique outperformed MEWS. The potential and accelerating power of ML in risk stratification and adverse event prediction might empower physicians to make precise decisions in the clinical environment.

Traditional methods, such as logistic regression, are the standard for developing prediction models. Over 20 types of early warning score systems have been developed for different populations to predict the chances of adverse events in patients with ED patients (Nannan Panday et al., 2017). Some of these systems showed better performance than MEWS in predicting in-hospital mortality, such as the National Early Warning Score (Nannan Panday et al., 2017), electronic cardiac arrest risk triage score (AUROC of 0.801 *vs.* 0.698) (Green et al., 2018), and rapid emergency medicine score (AUROC 0.707 *vs.* 0.630) (Bulut et al., 2014). It is difficult to compare our results with all other scores owing to the limited data availability and the retrospective nature of our study. Therefore, we only compared our results with MEWS, which is the most widely cited tool. In addition, these conventional statistical scoring systems have several common limitations, including generalizability, when applied to populations that differ from the derivation cohort (Stiell & Bennett, 2007), lack of the ability to update new variables (Pearce et al., 2006), and worse predictive accuracy during external validation (Siontis et al., 2015). Previous studies have shown that ML techniques are also better than conventional scoring systems in other fields, such as in patients with upper gastrointestinal bleeding (Shung et al., 2020) or chest pain (Liu et al., 2014).

ED physicians are challenged with an overwhelming diversity of tools, most of which have never been implemented or assessed for comparative effectiveness. Although our ML model showed better predictability than MEWS measured by AUROC and AUPRC, it is still not good enough and needs further investigation for practical clinical use. In addition, the risk of death is only a part of poor outcomes in the ED, and predictions of morbidity or disability risks are also important. Artificial intelligence (AI) may be the next major technological breakthrough that impacts healthcare delivery. Our study provides a good reference for any attempt to predict in-hospital mortality for non-traumatic ED patients in such a large number of cases. Hopefully, future research can further outperform our proposed method and approach more clinical values.

## Limitations

First, our goal was to compare the stacking ML model with other popular ML models and the MEWS scoring system in a standardized framework; we did not compare all available ML models and scoring systems or their variations. There are hundreds of different techniques and variations, and a comprehensive study is not feasible. Second, our prediction performance might be overestimated by missing patients who experienced significant deterioration after discharge or died at home or in other hospitals. We believe that the missing data were limited, as the overall event rate of the 72-h revisit with out-of-hospital cardiac arrest was as low as 6.6 per 100,000 discharges in our system. Third, patients suffering from out-of-hospital deaths after discharge from in-hospital care could not be traced in our database, so the long-term death predictions were probably less reliable. Fourth, the proposed ML models could not be used for patients with out-of-hospital death status, as these patients were excluded. Finally, there is a data imbalance problem with non-survival patients comprising <1% of the dataset. Although the proposed ML model shows better performance than MEWS, its value is still not ideal for clinical implications. Future research should improve the effectiveness of the ML model and whether it translates into improved clinical outcomes in patients with ED.

## Conclusions

In this multicenter study, using the AUROC and AUPRC metrics, we found that ML methods slightly improved the predictability of in-hospital mortality than MEWS in the prediction of early or delayed mortality. The use of prediction algorithms derived from ML techniques may improve the identification of patients with an increased risk of in-hospital mortality.

## Additional Information and Declarations

### Data Availability

The following information was supplied regarding data availability: The dataset is available at figshare: Wu, Kuan-Han (2020): PEERJ-AI-MEWS. figshare. Dataset. https://doi.org/10.6084/m9.figshare.13033028.v1.
